# *Arcobacteraceae* are ubiquitous mixotrophic bacteria playing important roles in carbon, nitrogen, and sulfur cycling in global oceans

**DOI:** 10.1128/msystems.00513-24

**Published:** 2024-06-21

**Authors:** Jianyang Li, Shizheng Xiang, Yufei Li, Ruolin Cheng, Qiliang Lai, Liping Wang, Guizhen Li, Chunming Dong, Zongze Shao

**Affiliations:** 1 Key Laboratory of Marine Genetic Resources, Key Laboratory of Marine Genetic Resources of Fujian Province, Third Institute of Oceanography, Ministry of Natural Resources of PR China, Xiamen, China; 2 Southern Marine Science and Engineering Guangdong Laboratory (Zhuhai), Zhuhai, China; Oregon State University, Corvallis, Oregon, USA

**Keywords:** *Arcobacteraceae*, mixotrophy, carbon fixation, sulfur oxidation, methane oxidation, denitrification

## Abstract

Mixotrophy is an important trophic strategy for bacterial survival in the ocean. However, the global relevance and identity of the major mixotrophic taxa remain largely elusive. Here, we combined phylogenetic, metagenomic, and metatranscriptomic analyses to characterize ubiquitous *Arcobacteraceae* based on our deep-sea *in situ* incubations and the global data. The phylogenomic tree of *Arcobacteraceae* is divided into three large clades, among which members of clades A and B are almost all from terrestrial environments, while those of clade C are widely distributed in various marine habitats in addition to some terrestrial origins. All clades harbor genes putatively involved in chitin degradation, sulfide oxidation, hydrogen oxidation, thiosulfate oxidation, denitrification, dissimilatory nitrate reduction to ammonium, microaerophilic respiration, and metal (iron/manganese) reduction. Additionally, in clade C, more unique pathways were retrieved, including thiosulfate disproportionation, ethanol fermentation, methane oxidation, fatty acid oxidation, cobalamin synthesis, and dissimilatory reductions of sulfate, perchlorate, and arsenate. Within this clade, two mixotrophic Candidatus genera represented by UBA6211 and CAIJNA01 harbor genes putatively involved in the reverse tricarboxylic acid pathway for carbon fixation. Moreover, the metatranscriptomic data in deep-sea *in situ* incubations indicated that the latter genus is a mixotroph that conducts carbon fixation by coupling sulfur oxidation and denitrification and metabolizing organic matter. Furthermore, global metatranscriptomic data confirmed the ubiquitous distribution and global relevance of *Arcobacteraceae* in the expression of those corresponding genes across all oceanic regions and depths. Overall, these results highlight the contribution of previously unrecognized *Arcobacteraceae* to carbon, nitrogen, and sulfur cycling in global oceans.

**IMPORTANCE:**

Marine microorganisms exert a profound influence on global carbon cycling and ecological relationships. Mixotrophy, characterized by the simultaneous utilization of both autotrophic and heterotrophic nutrition, has a significant impact on the global carbon cycling. This report characterizes a group of uncultivated bacteria *Arcobacteraceae* that thrived on the “hot time” of bulky particulate organic matter and exhibited mixotrophic strategy during the *in situ* organic mineralization. Compared with clades A and B, more unique metabolic pathways were retrieved in clade C, including the reverse tricarboxylic acid pathway for carbon fixation, thiosulfate disproportionation, methane oxidation, and fatty acid oxidation. Global metatranscriptomic data from the Tara Oceans expeditions confirmed the ubiquitous distribution and extensive transcriptional activity of *Arcobacteraceae* with the expression of genes putatively involved in carbon fixation, methane oxidation, multiple sulfur compound oxidation, and denitrification across all oceanic regions and depths.

## INTRODUCTION

Marine microorganisms exert a profound influence on global carbon cycling and ecological dynamics within the ocean’s interior, not only through the conversion of organic material from surface-dwelling phytoplankton via a heterotrophic strategy but also through the chemoautotrophic fixation of dissolved inorganic carbon (DIC) (1). Mixotrophy, characterized by the simultaneous utilization of both autotrophic and heterotrophic nutrition (2), has emerged as a potentially pivotal trophic strategy among planktonic microbes in the open ocean, and its integration into microbial loop models is particularly crucial (3, 4). The effects of mixotrophy on community and ecosystem dynamics are now unclear, but it has been postulated that it could enhance DIC fixation, elevate the transfer of organic matter to higher trophic levels, increase nutrient retention within ecosystems, and amplify the effectiveness of biological carbon pumps (3, 5). Nevertheless, despite the widespread occurrence of mixotrophy in the ocean (2), such as certain members within SAR324, SUP05, and UBA868 (1, 6–8), the relevant taxa and energy selection strategies supporting DIC fixation remain enigmatic (9).

The energy required for DIC fixation can be obtained from the oxidization of diverse inorganic molecules, including ammonia, nitrite, hydrogen, and reduced sulfur compounds. Crenarchaeota Group I, known as ammonium-oxidizing archaea, represents the most abundant chemoautotrophs in the dark ocean (10, 11). Additionally, recognized marine nitrite-oxidizing bacteria encompass the phyla *Nitrospinae* and *Nitrospirae* (6). Nevertheless, neither ammonium nor nitrite oxidation alone proves sufficient to support the observed rates of carbon fixation beyond the oxygen minimum zones (OMZs) of the global ocean (6, 12, 13).

Oxidation of reduced sulfur compounds stands out as a major inorganic chemical energy source for DIC fixation. This process is typically prevalent in hydrothermal vent plumes and OMZs, where hydrogen sulfide plumes recurrently erupt (14–17). Similarly, within locally organic-rich and anoxic ecosystems formed around whale falls, wood falls, bone falls, and kelp falls, microorganisms generate a substantial quantity of reduced sulfur compounds through sulfate reduction (18). These compounds, in turn, support a thriving population of sulfur-oxidizing bacteria (SOBs) (19, 20).

Our recent deep-sea *in situ* organic matter enrichment (DIME) study revealed that bulky organic matter-supported bacterial communities contained a large number of SOBs, including members of *Campylobacter*, such as *Arcobacteraceae*, *Sulfurospirillaceae*, and *Sulfurovaceae*, in addition to bacteria of the families *Marinifilaceae* and *Vibrionaceae*, both of which play crucial roles in organic matter decomposition (21, 22). Particularly noteworthy, members of the family *Arcobacteraceae* were found to be predominant in all our DIME communities across different oceans, including the Pacific Ocean, the South China Sea, and the Indian Ocean (21). Although members of the *Arcobacteraceae* family have been primarily associated with gastrointestinal pathogens since 1991 (23–25), they are frequently encountered in diverse habitats, including food and food-processing facilities, underground water, surface water, sewage, and seawater (26–29), even in chemosynthetic ecosystems of hydrothermal vents and wood and whale falls (19, 20). It is unclear whether *Arcobacteraceae* are chemolithotrophs, chemoorganotrophs, or mixotrophs in these deep-sea chemosynthetic ecosystems.

In this study, we conducted phylogenetic, metagenomic, and metatranscriptomic analyses to specify the genetic diversity and metabolic characteristics of *Arcobacteraceae* by encompassing both the members that dominate our DIME communities and those that predominate in deep-sea *in situ* communities on sinking particulate organic matter collected by sediment traps at abyssal depths of ALOHA (28). Furthermore, we conducted a global survey to assess the expression of key genes involved in carbon, nitrogen, and sulfur metabolism within this family across all samples collected during the Tara Oceans expedition. These integrated analyses are anticipated to significantly expand our understanding of the trophic strategies and ecological roles of the widespread *Arcobacteraceae* in the global environment, particularly in marine ecosystems.

## RESULTS AND DISCUSSION

### The potential of sulfur oxidation and DIC fixation of *Arcobacteraceae* as a dominant member in deep-sea communities supported by organic matter

**Fig 1:**
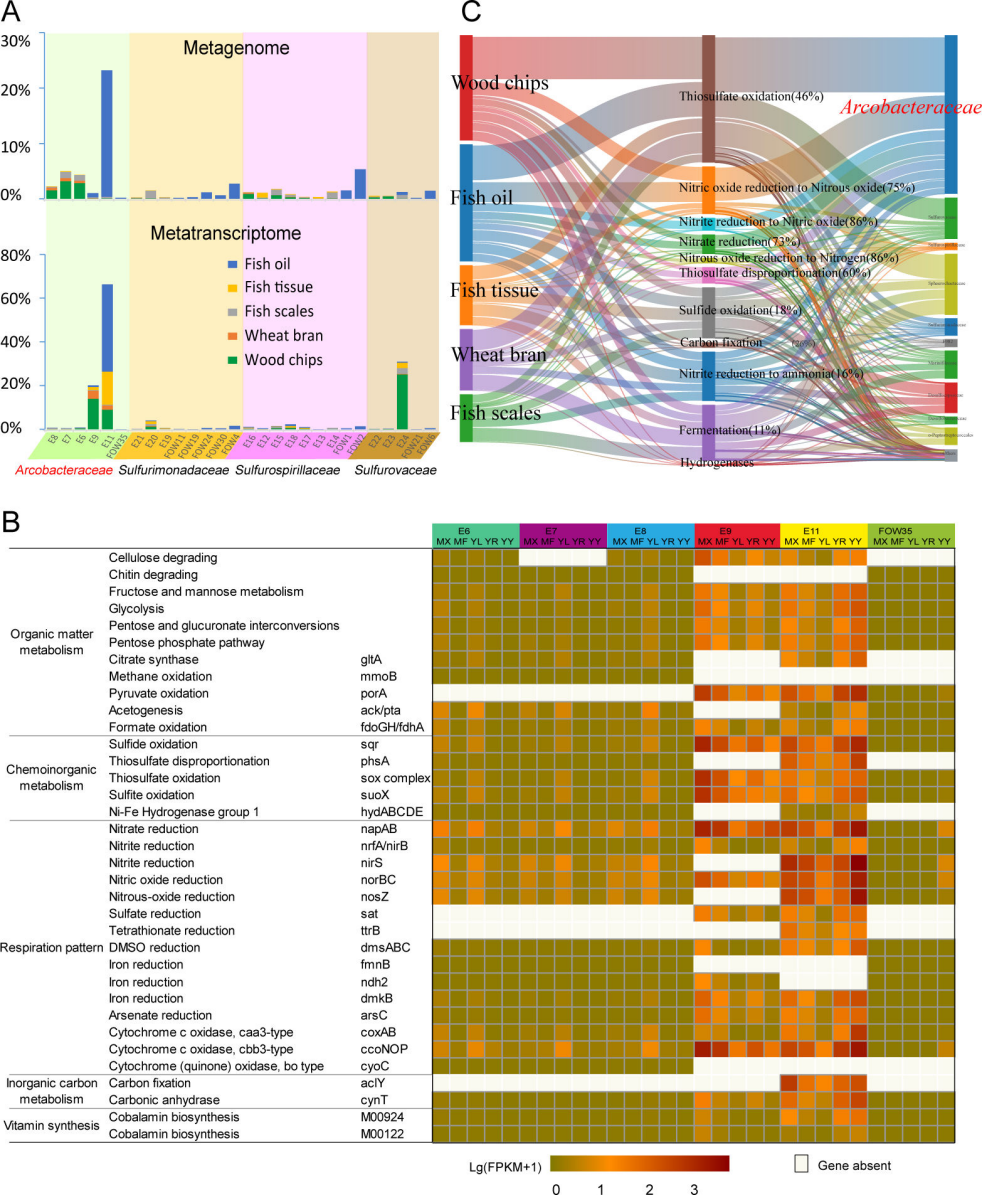
Transcriptional profiles of preponderant *Arcobacteraceae* in deep-sea communities supported by organic matters. (**A**) Percentage of *Campylobacter* MAGs, including *Arcobacteraceae*, *Sulfurimonadaceae*, *Sulfurospirillaceae*, and *Sulfurovaceae*, in the deep-sea assemblages amended with wood chips, wheat bran, fish scales, fish tissue, and fish oil based on metagenomic (top) and metatranscriptomic (bottom) data sets. (**B**) Metabolic transcriptional profiles of nine *Arcobacteraceae* MAGs associated with organic matter metabolism, chemoinorganic metabolism, respiration, and carbon fixation in these five assemblages enriched with wood chips (MX), wheat bran (FP), fish scales (YL), fish tissue (YR), and fish oil (YY). (**C**) Transcriptional distribution patterns of gene sets or metabolism pathways related to sulfur compound oxidation or disproportionation, carbon fixation, fermentation, dissimilatory nitrate reduction to ammonium (DNRA), denitrification, and hydrogen oxidation in different MAGs in the above five enriched consortia. The percentage in brackets refers to the proportion of *Arcobacteraceae* in the corresponding metabolic or genetic transcript.

In our recent report, a large number of *Arcobacteraceae* were detected in deep-sea *in situ* enrichments with natural organic matter in pelagic areas, including stations on a flat-topped seamount in the western Pacific Ocean (1,622 m water depth and 2.44°C), on the seafloor in the South China Sea (3,758 m water depth and 2.39°C), and in the deep-sea basin beside the southwest Indian Ridge in the Indian Ocean (4,434 m water depth and 2.35°C) (21, 30). In this study, we obtained six high-quality metagenome-assembled genomes (MAGs) (E6–E9, E11, and FOW35) belonging to *Arcobacteraceae* from DIME metagenomic data to study their trophic strategies and ecological roles *in situ* (Table 1). Among them, MAGs E9 and E11 were particularly prominent in the five DIME communities, as was their sister family member MAG E24 based on metatranscriptomic data (Fig. 1A; Table S1). These two MAGs accounted for 4% and 13% of the metatranscriptomic data (Table S1), respectively, making them the second most transcriptionally active members within the consortia, following the predominant *Marinifilaceae* that function as macromolecule depolymerizers (30). Their predominance in these organic matter-supported chemosynthetic consortia regardless of organic matter types and oceanic areas implies an essential role during the process of *in situ* organic mineralization.

**TABLE 1:** Genomic features of *Arcobacteraceae* MAGs obtained in this study

MAGs	Source^*a*^	Genus^*b*^	Completeness (%)	Contamination (%)	GC content (%)	N50 (bp)	Genomic size (bp)	No. of predicted genes	EggNOG (%)	KEGG (%)
E9	DIMEs	CAIJNA01	95.61	1.321	27.7	13,538	2,363,063	2,288	1,941 (84.8)	1,443 (63.1)
E11	DIMEs	CAIJNA01	98.27	1.829	26.7	35,255	3,671,745	3,582	2,914 (81.4)	1,952 (54.5)
mgm_14	Wood falls	CAIJNA01	99.59	2.845	31.8	91,191	3,005,364	2,918	2,537 (86.9)	1,482 (50.8)
G04.16	SPOMs	NORP36	99.18	1.964	28.7	178,853	3,209,010	3,088	2,654 (85.9)	1,658 (53.7)
G08.16	SPOMs	NORP36	97.96	2.235	25.7	110,966	3,217,814	2,992	2,432 (81.3)	1,566 (52.3)
G09.16	SPOMs	NORP36	92.59	2.953	30.9	6,698	3,384,932	3,727	2,944 (79.0)	1,785 (47.9)
G10.12	SPOMs	NORP36	76.30	1.724	26.7	7,044	3,024,018	3,332	2,622 (78.7)	1,643 (49.3)
mgm_04	Wood falls	*Arcobacter*	96.56	2.439	27.6	13,594	2,687,186	2,874	2,540 (88.4)	1,703 (59.3)
E6	DIMEs	*Halarcobacter*	99.59	2.439	27.0	187,762	3,487,880	3,361	2,942 (87.5)	2,068 (61.5)
E7	DIMEs	*Halarcobacter*	98.78	2.642	27.0	256,231	3,483,567	3,325	2,912 (87.6)	2,040 (61.4)
E8	DIMEs	*Halarcobacter*	99.79	2.032	27.1	48,776	3,495,879	3,450	2,049 (59.4)	2,079 (60.3)
FOW35	DIMEs	*Poseidonibacter*	78.08	3.570	27.3	17,830	2,319,618	2,451	2,145 (87.5)	1,502 (61.3)
mgm_09	Wood falls	Unclassified clade	90.53	4.674	28.5	25,363	3,352,549	3,371	2,673 (79.3)	1,610 (47.8)
mgm_17	Wood falls	Unclassified clade	89.09	3.658	30.2	26,262	3,789,136	3,723	2,931 (78.7)	1,732 (46.5)
mgm_23	Wood falls	Unclassified clade	71.79	1.761	30.2	3,095	2,539,868	3,085	2,255 (73.1)	1,384 (44.9)

^*a*^ DIME, deep-sea *in situ* organic matter enrichment; SPOM, sinking particulate organic matter collected by sediment traps at ALOHA.

^*b*^ Taxonomic assessment of each MAG was performed using GTDBTk v2.2.3 with the database release 207.

Subsequent genomic analysis revealed that *Arcobacteraceae* in the DIME communities possesses a range of organic carbon metabolism putative capabilities, including cellulose and chitin hydrolysis and the ability to oxidize various small organic molecules, such as pyruvate (*porA*), formate (*fdoGH*/*fdhA*), and methane (*mmoB*) (Fig. 1B; Fig. S1). These metabolic pathways, especially those involved in the small molecule organic metabolism, were corroborated by metatranscriptomic data (Fig. 1B), suggesting that *Arcobacteraceae* may mainly utilize small organic molecules such as monosaccharides and organic acids generated from depolymerization and hydrolysis of macromolecules and fermentation by the pioneer bacteria of *Marinifilaceae* (30) and *Vibrionaceae* (22).

**Fig 2:**
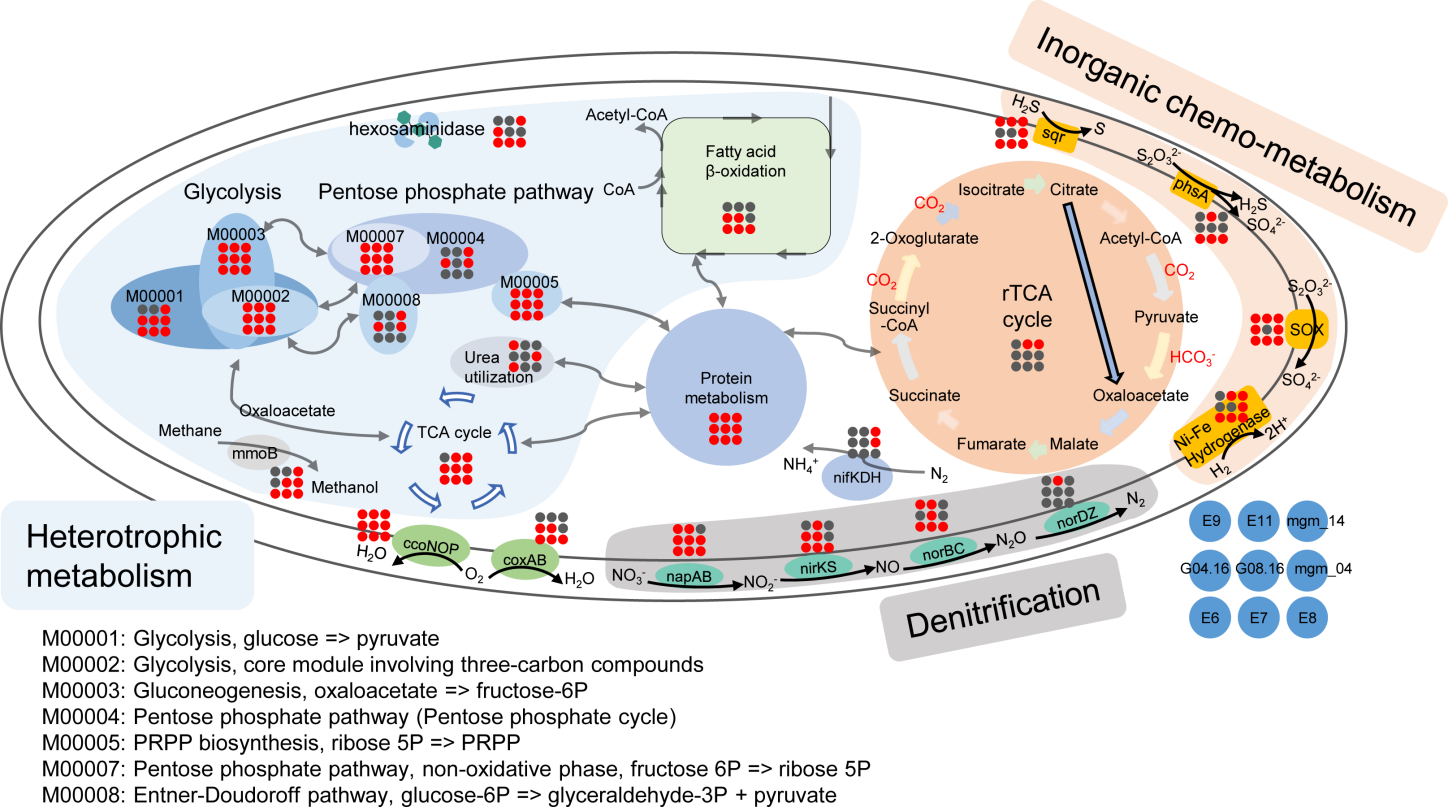
Metabolic capabilities of the *Arcobacteraceae* MAGs obtained from the five DIME samples, 63 sinking particulate organic matter (SPOM) samples, and wood falls. Pathways associated with heterotrophic metabolism including glycolysis, pentose phosphate, urea utilization, methane oxidation, and fatty acid β-oxidation; inorganic chemo-metabolism including thiosulfate oxidation, thiosulfate disproportionation, sulfide oxidation, and hydrogen oxidation; and denitrification are shown. Genes appearing in corresponding genome are shown in red, and genes absent are shown in black. MAGs E6–E9 and E11 were obtained from the five DIME samples, G04.16 and G08.16 were obtained from 63 SPOM samples, and mgm_14 and mgm_04 were obtained from wood falls.

Additionally, the gene encoding ATP-dependent citrate lyase (*aclY*, alpha-subunit, and beta-subunit) involved in the reverse tricarboxylic acid (rTCA) cycle for DIC fixation (31) was retrieved in *Arcobacteraceae* MAG E11 (Fig. 1B; Fig. S2). Metatranscriptomic data further highlighted their significance in performing both heterotrophic and autotrophic metabolisms during organic mineralization in the deep sea. *Arcobacteraceae* is the second contributor to the transcript of the *aclY* gene (*Desulfocapsaceae* is the first contributor, data not shown), accounting for 26% of all *aclY* transcripts in our DIME communities (Fig. 1C). The *aclY* transcript from *Arcobacteraceae* is much higher than that in its sister family *Sulfurimonadaceae* (analysis of variance [ANOVA], Tukey’s test, *P* < 0.05). The above results suggest that bacterium E11 belongs to mixotrophic microorganisms and performs both inorganic autotrophic and organic heterotrophic processes during the organic mineralization *in situ* (Fig. 1B and 2).

Regarding inorganic chemometabolisms for energy requirements, most members of *Arcobacteraceae* in our DIME communities exhibit the putative ability to oxidize various inorganic compounds by encoding genes for thiosulfate oxidation (*Sox* complex) (Fig. S3 and S4), sulfide oxidation (*sqr*) (Fig. S5), thiosulfate disproportionation (*phsA*) (Fig. S6), and hydrogen oxidation (NiFe-group1 and NiFe-group3) (Fig. 1B). Previous studies have found that some members of *Arcobacteraceae* can oxidize hydrogen sulfide (23, 32). Moreover, gene expression quantification in our DIME consortia showed that 18%, 46%, and 60% of transcripts assigned to sulfide oxidation, thiosulfate oxidation, and thiosulfate disproportionation, respectively, were generated from *Arcobacteraceae* (Fig. 1C). These results indicated that *Arcobacteraceae* was a major group in inorganic sulfur compound oxidation in our DIMEs. In turn, these compounds could provide energy for *Arcobacteraceae* growth yields, in addition to the organic matter mentioned above (Fig. 1B and 2). Sulfide-oxidizing bacteria activity (oxidizing sulfide coupled with denitrification) could gain a high amount of energy (ΔG’ = −468 to −661 kJ mol^−1^) under OMZ conditions, while sulfate-reducing bacteria gain a moderate amount of energy (ΔG’ = −88 kJ mol^−1^) by using acetate as the electron donor coupling with the reduction of sulfate to sulfide (33). Moreover, laboratory growth yield results showed that the biomass yield by microbial oxidation inorganic sulfur compounds (mol^−1^) for DIC fixation was higher than that from the oxidation of small molecules of organic matters such as acetic acid, formic acid, and ethanol under anaerobic conditions (34).

With respect to electron receptors coupling substrate oxidation, *Arcobacteraceae* possess versatile putative anaerobic respiration pathways (Fig. 1B and 2), including DNRA (*napAB*), denitrification (*nirS*, *norBC*, and *nosZ*), DMSO reduction (*dmsABC*), Fe^3+^ reduction (*fmnB*, *ndh2*, and *dmkB*), and arsenate reduction (*arsC*). The ability of dissimilating nitrate reduction for *Arcobacteraceae* has been demonstrated in laboratory experiments, such as *Arcobacter lacus* RW43-9^T^ and *Arcobacter caeni* RW17-10^T^ (24). Metatranscriptomic data in our DIMEs showed that transcripts of genes putatively associated with denitrification and DNRA pathways in *Arcobacteraceae* were expressed *in situ*, which shared approximately three-quarters of the corresponding transcripts (Fig. 1C). These results suggest that *Arcobacteraceae* is involved in nitrogen cycling during *in situ* organic mineralization in the deep ocean. Among them, members represented by MAG E9 and E11 are the most critical representatives in the processes (Fig. 1B and 2).

Similarly, at the abyssal depths of ALOHA, Boeuf et al. observed *Arcobacteraceae* as one of the most predominant members in the SPOM collected by sediment traps, indicating its central role in the mineralization and biogeochemical transformation of SPOM in the deep ocean (28). To make a comparison with ours in DIME, we reassembled and retrieved four *Arcobacteraceae* MAGs (G04.16, G08.16, G09.16, and G10.12) from the metagenomic data sets of ALOHA (Table 1). SPOM metatranscriptomic analysis revealed that *Arcobacteraceae* was the most active microorganism at ALOHA, which did dominate the transcripts putatively related to denitrification, DNRA, and thiosulfate oxidation, as well as fermentation (ethanol production and acetate production [*acdA*, *ack*, and *pta*]) (Fig. S7). However, no gene encoding *aclY* or other known genes involved in DIC fixation were retrieved in these *Arcobacteraceae* genomes (Fig. 2). We speculated that they may have an unknown DIC fixation pathway or, through a stealth reaction, the roTCA pathway driving DIC fixation (35, 36). Regardless, they retain energy draining from inorganic compounds to a certain degree in the deep water column.

Consistent with our findings, a large number of *Arcobacteraceae* were also found in chemosynthetic ecosystems rich in organic matter, such as unique deep sea ecosystems of whale falls (20), wood falls (19), and bones (37). In the phylogenetic tree based on 16S rRNA gene sequences, these *Arcobacteraceae* were clustered into closely related branches (21). This corroborates that the occurrence of *Arcobacteraceae* in organic-rich niches is not coincidental but rather a result of natural selection, contributing to the establishment and maintenance of the chemosynthetic ecosystem in the deep sea. Reduced sulfur compounds, such as sulfide and thiosulfate produced by sulfate reduction driven by organic mineralization in both our artificial natural organic falls and wood falls in the deep sea, can serve as the energy source and be restored by a series of bacteria belonging to the phylum *Campylobacter* via sulfide oxidation and thiosulfate oxidation used to fix DIC, thereby avoiding energy escape from the organic matter-supported ecosystem in the extremely oligotrophic deep sea. Similarly, chemoautotrophic nitrifiers were found to fix DIC and produce a series of new organic compounds to maintain community metabolism through particulate organic matter degradation, particularly with nitrogenous compounds, indicating that nitrifiers may play an important role in the processes of particulate organic matter transformation and remineralization in the aphotic ocean layer (38). The new organic carbon molecules produced by chemolithotrophs in the dark ocean have drawn cumulative attention and are probably high relative to the organic carbon supplied by sinking particles (39).

### Phylogeny, niche partitioning, and metabolic adaptation of *Arcobacteraceae*

**Fig 3:**
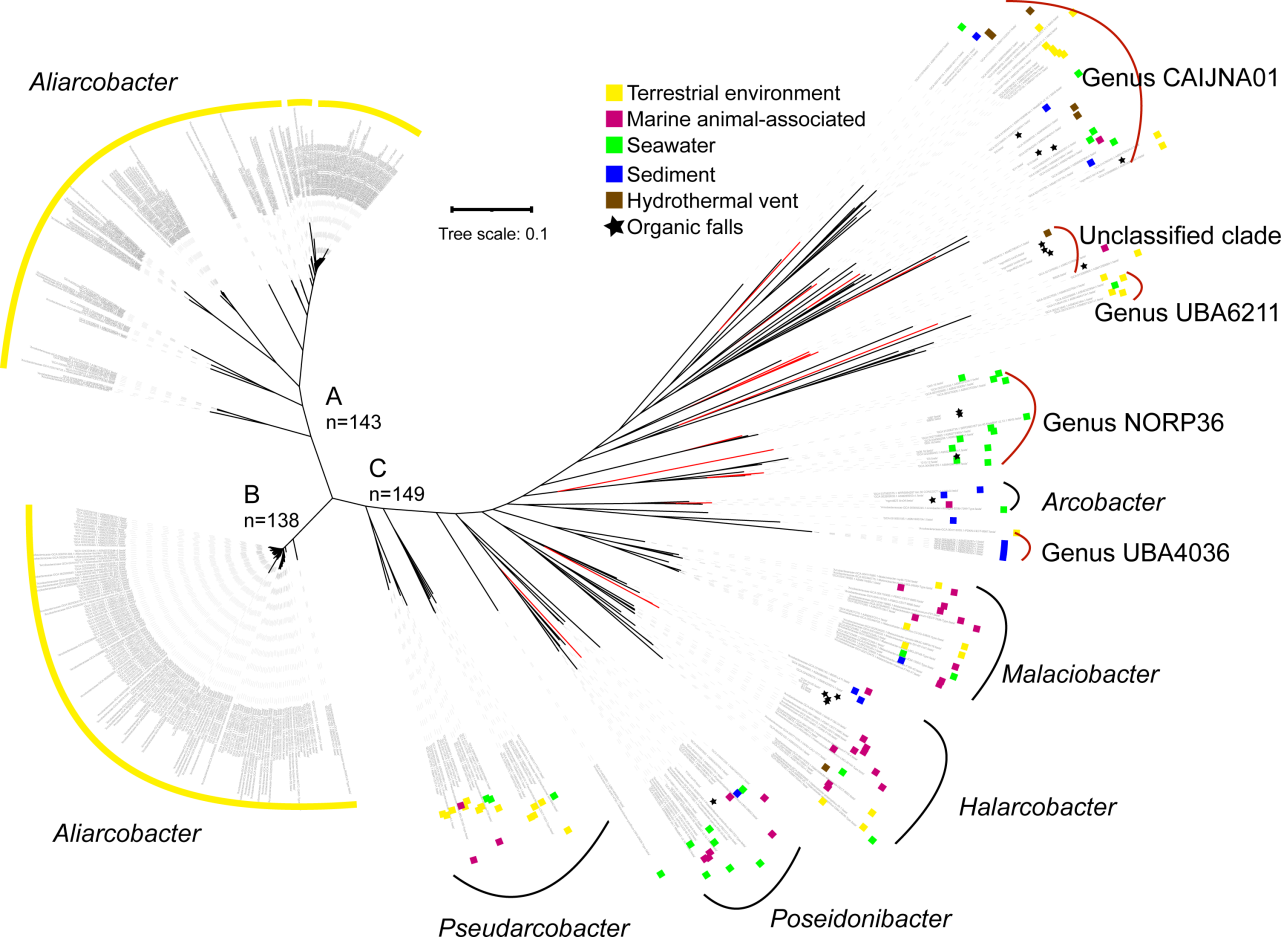
Phylogenomics and habitat distribution of *Arcobacteraceae*. Phylogenomic tree constructed from 92 concatenated core genes of the family *Arcobacteraceae*, employing the up-to-date bacterial core gene (UBCG) method (40). A total of 430 *Arcobacteraceae* genomes (cultured and uncultured bacteria) with completeness  > 70% and contamination < 10% were obtained. The red branches represent *Arcobacteraceae* MAGs from our study. The members of clades A and B are almost all terrestrial in origin and are mainly associated with human and animal diseases. Clade C members showcase diverse marine habitats, including seawater, sediment, hydrothermal environment, whale and wood falls, and other habitats associated with marine animals in addition to certain terrestrial environments such as sewage, sulfidic caves, and lakes.

To gain a deeper understanding of the niche partitioning and ecological roles of *Arcobacteraceae* in the global oceans, we conducted a comprehensive analysis encompassing all members of this family. For this, a phylogenomic analysis was conducted by recruiting publicly available *Arcobacteraceae* genomes (including cultured and uncultured bacteria), in addition to our six MAGs from DIME communities, four from SPOM at ALOHA, and five from deep-sea wood falls on a shelf (Table 1). The resulting phylogenomic tree revealed that *Arcobacteraceae* differentiated into three major clades, namely, A, B, and C. Notably, clades A and B were exclusively composed of the sole genus *Aliarcobacter*, and intriguingly, almost all members within these two clades were originally from terrestrial environments (Table S2; Fig. 3), including human and animal intestines (25, 27). In stark contrast, members of clade C exhibited a much wider distribution across diverse habitats in marine ecosystems, including seawater, sediment, hydrothermal environments, whale and wood falls, sewage, sulfidic caves, lakes, and other habitats associated with marine animals (Table S2; Fig. 3). This clade encompasses a series of genera of *Arcobacteraceae*, such as *Malaciobacter*, *Halarcobacter*, *Poseidonibacter*, *Pseudarcobacter*, and *Arcobacter*, in addition to uncultured species that have yet to be classified (Fig. 3). Among the unclassified species, at least five potential novel genera were formed according to the results of species annotation based on the Genome Classification Database (GTDB) (Fig. 3). In the tree, the Candidatus genus, represented by the bacterium of MAG CAIJNA01 that originated from a lake, contained the species of MAGs E9 and E11, in addition to five hydrothermal-origin MAGs; four MAGs from the SPOM of the water column at ALOHA and one MAG from the marine bone-degrading microbiome were assigned to the Candidatus genus represented by NORP36 that was from marine sediment at a depth of 4400 m; additionally, three MAGs obtained from the wood-fall samples were clustered closely and possibly belonged to three genera without represented MAGs available in the GTDB (Fig. 3); the members of the Candidatus genus UBA6211 are mainly derived from the sewage environment. These results indicate that *Arcobacteraceae* have been differentiated in accordance with their habitats. Niche partitioning possibly occurs along with environmental adaptations within the family, as further detailed below.

**Fig 4:**
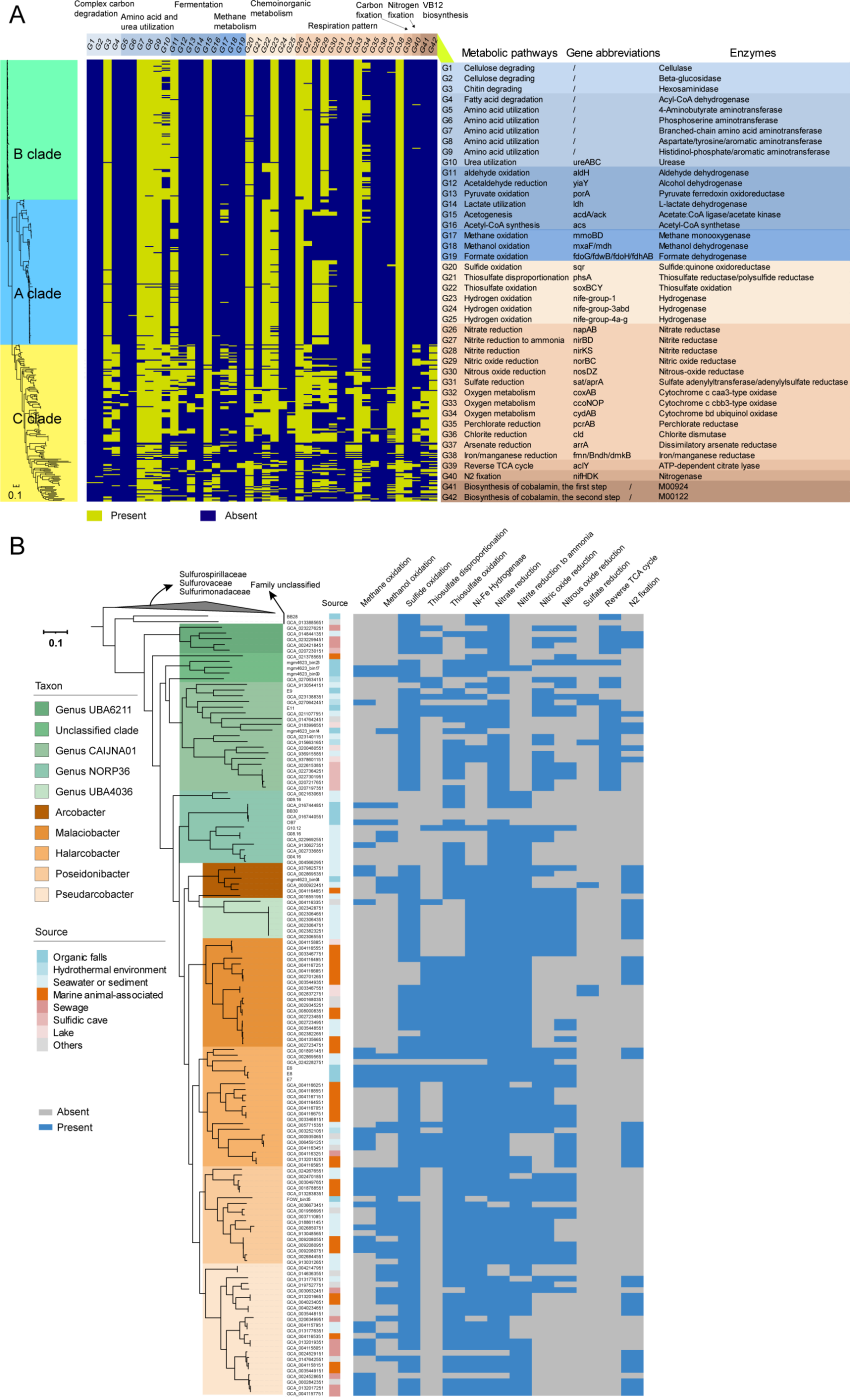
Metabolic variation within the family *Arcobacteraceae*. (**A**) Distinct metabolic pathways across clades A, B, and C. “/” indicates that there are currently no gene abbreviations; M00924 and M00122 are two steps involved in the anaerobic synthesis of cobalamin (VB12), namely, the first step for corrin ring biosynthesis and the second step for cobalamin biosynthesis from cobyrinic acid. (**B**) Metabolic differentiation within clade C, with a phylogenomic tree constructed using the UBCG method. The families *Sulfurimonadaceae*, *Sulfurospirillaceae*, and *Sulfurovaceae* were utilized as reference outgroups. Unidentified or uncultivable MAGs were taxonomically classified via the GTDB database.

Furthermore, to gain insights into the differentiation within the family, the metabolic features unique in certain clades or shared among different clades were analyzed based on genomic comparison. The results showed that all the clades harbored genes with the potential for chitin degradation, sulfide oxidation, hydrogen oxidation, thiosulfate oxidation, denitrification, DNRA, microaerophilic respiration, and metal (iron/manganese) reduction (Fig. 4A). The three clades (A to C) shared numerous common metabolic pathways, but more divergent pathways putatively involved in carbon, nitrogen, and sulfur cycling were uniquely found in clade C (Fig. 4A). For instance, in terms of the metabolism of simple organic molecules, genes putatively related to ethanol fermentation, pyruvate oxidation, methanol oxidation, formate oxidation, and fatty acid oxidation metabolism were merely retrieved in the genomes of clade C. Furthermore, members of this clade harbor the potential for thiosulfate disproportionation, DIC fixation, nitrogen fixation, and the cobalamin (VB_12_) synthesis, which are absent in the other two clades, except for two genomes in clade A containing the nitrogen fixation pathway (Fig. 4A). Moreover, members of clade C display enhanced versatility in anaerobic respiration compared with the other two clades, including reduction of sulfate, perchlorate, and arsenate in addition to denitrification and DNRA (Fig. 4A). Therefore, members of clade C may have stronger environmental adaptability than the other two clades, which perfectly matched the survey of its distribution in wide ecological environments.

Within clade C, the rTCA DIC fixation pathway was primarily found in the Candidatus genus UBA6211, which comprises three sewage MAGs, and the Candidatus genus CAIJNA01, which contains our DIME MAGs E9 and E11, along with five hydrothermal MAGs (Fig. 4B). This finding indicates their potential for DIC fixation via chemoautotrophy in addition to organic carbon utilization (Fig. 4B). These two novel genera thus belonged to the mixotrophic microorganisms, and they mainly use reduced sulfur compounds and hydrogen as inorganic chemical energy for DIC fixation, coupled with nitrate reduction (Fig. 4B). The cultivable strain *Arcobacter* sp. FWKO B (GCA_014844135.1, located within the Candidatus genus UBA6211 in the phylogenomic tree) isolated from produced brine at the Coleville oil field has been demonstrated to utilize hydrogen, hydrogen sulfide, and formate as energy sources and exhibits chemolithoautotrophic growth in the presence of elemental sulfur, hydrogen, and DIC, coupled with nitrate reduction to nitrite (32).

Although *aclY* was not detected in most branches of clade C, most species possessed genes putatively related to the oxidation of reduced sulfur compounds and hydrogen (Fig. 4B), which would allow them to derive energy from the oxidation of thiosulfate, sulfide, and hydrogen in addition to organic matter. Notably, the *aclY* gene and other DIC fixation pathways were not found in the genome of *Halarcobacter anaerophilus* IR-1, even though it could grow chemolithoautotrophically on hydrogen and hydrogen sulfide, in addition to organoheterotrophic growth on yeast extract, peptone, and various organic acids (41). This suggests the presence of another novel DIC fixation pathway in this mixotrophic bacterium. Similarly, *Alcanivorax* has been found to fix DIC through iron oxidation without any unknown DIC fixation pathway in its genomes (42), even though it is traditionally considered a heterotrophic bacterium. Therefore, we cannot rule out the possibility that *Arcobacteraceae* strains that contain no previously described DIC fixation pathways are actually mixotrophic, such as the *Arcobacter* collected from abyssal depths with sediment traps at ALOHA station (28), which warrants further investigation to confirm its capacity for DIC fixation.

### The roles of *Arcobacteraceae* in the cycling of carbon, nitrogen, and sulfur in the global oceans

The above results indicated that at least some members of *Arcobacteraceae* are mixotrophic microorganisms, carrying out DIC fixation through sulfide and thiosulfate oxidation or thiosulfate disproportionation coupled with DNRA and denitrification, highlighting their roles in global carbon, nitrogen, and sulfur cycling. To consolidate this hypothesis, we surveyed the distribution of *Arcobacteraceae* in global marine environments and the transcription of related metabolic genes therein.

**Fig 5:**
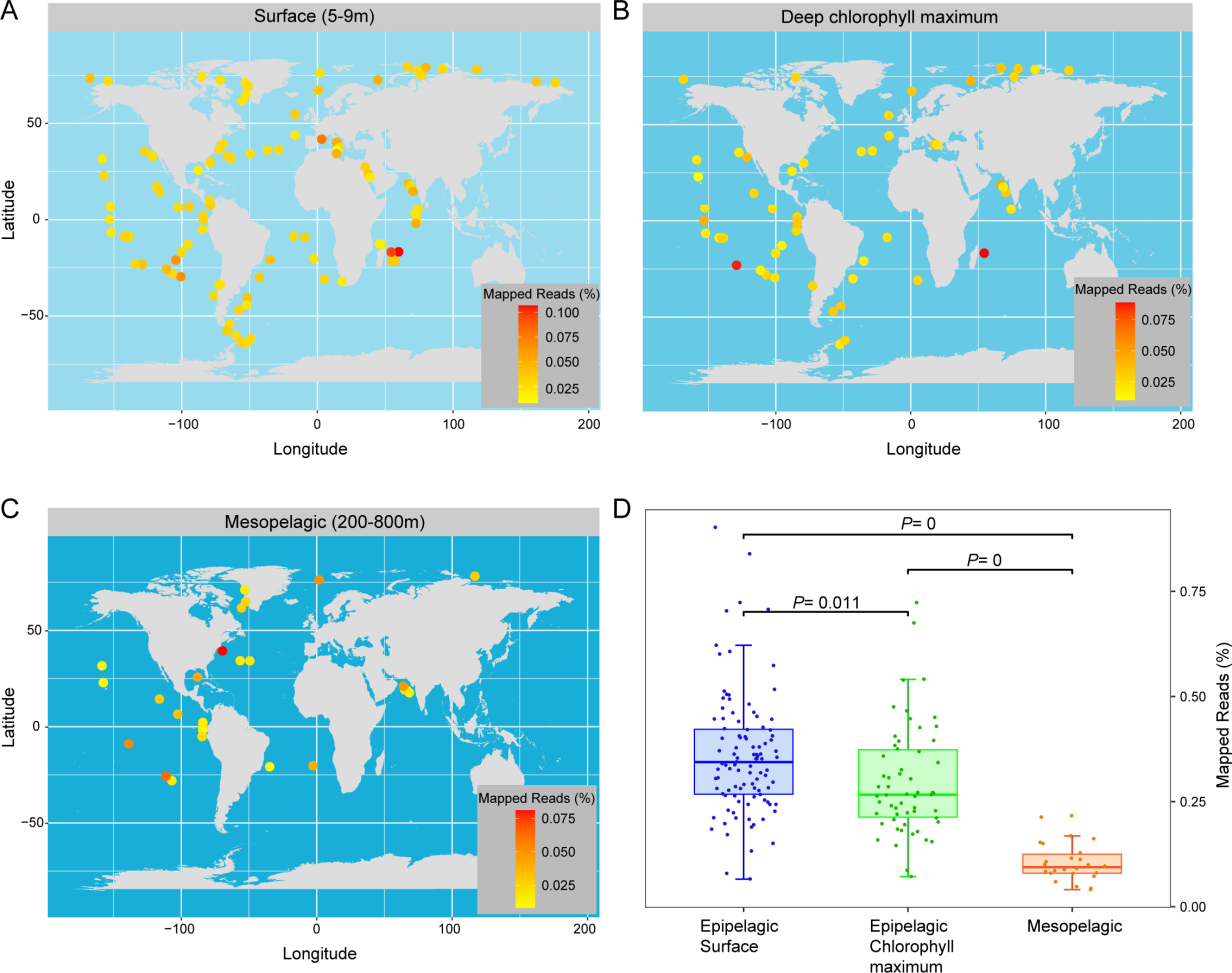
Global oceanic distribution pattern of *Arcobacteraceae*. (**A–C**) A visual representation of the metatranscriptomic reads mapped to *Arcobacteraceae* genomes, distributed across surface (**A**), deep chlorophyll maximum (**B**), and mesopelagic (**C**) layers. In this study, we mapped the metatranscriptomic reads from 187 Tara Oceans expedition sites to 82 de-redundant genomes of clade C. Each data point indicates a specific station or sample, with colors reflecting average percentage values. (**D**) Significant difference in the transcriptional activity of *Arcobacteraceae* between different water layers in the ocean. ANOVA and the post hoc Tukey HSD test were used for significant difference comparison.

First, we mapped the metatranscriptomic reads from 187 sites in the Tara Ocean to 82 de-redundant genomes (95% average nucleotide identity threshold) of clade C. The results showed that *Arcobacteraceae* was ubiquitous in all oceanic survey regions regardless of water depth (Fig. 5A through C) and its activity in the upper water column was significantly higher than that in the deep sea (ANOVA, Tukey’s test, *P* < 0.05; Fig. 5D). The number of reads mapped to each *Arcobacteraceae* genome accounted for an average of 0.025% in each sample (Table S3), which was comparable to that of SAR324 (the corresponding ratio of SAR324-2D in the mesopelagic layer was approximately 0.025%, and the ratio of SAR324-2A in the surface layer was approximately 0.08%) that ranks as one of the most frequently found and abundant bacterial members of deep ocean communities (43). In addition, in order to compare at the abundance level with the recently discovered ubiquitous mixotrophic bacterial group UBA868 in the marine environment, we used the same abundance standardization RPKG method (8). The results showed that the average RPKG values of *Arcobacteraceae* in the surface layer (5–9 m), deep chlorophyll maximum (DCM) layer, and mesopelagic layer (200–800 m) were 0.6, 0.45, and 0.3, respectively, which were comparable to that of UBA868 (approximately 0.2–0.5 in the mesopelagic layer) (8). These results indicate that *Arcobacteraceae* are active and play a nonnegligible role in element cycling in the marine water column, especially in the upper water column.

**Fig 6:**
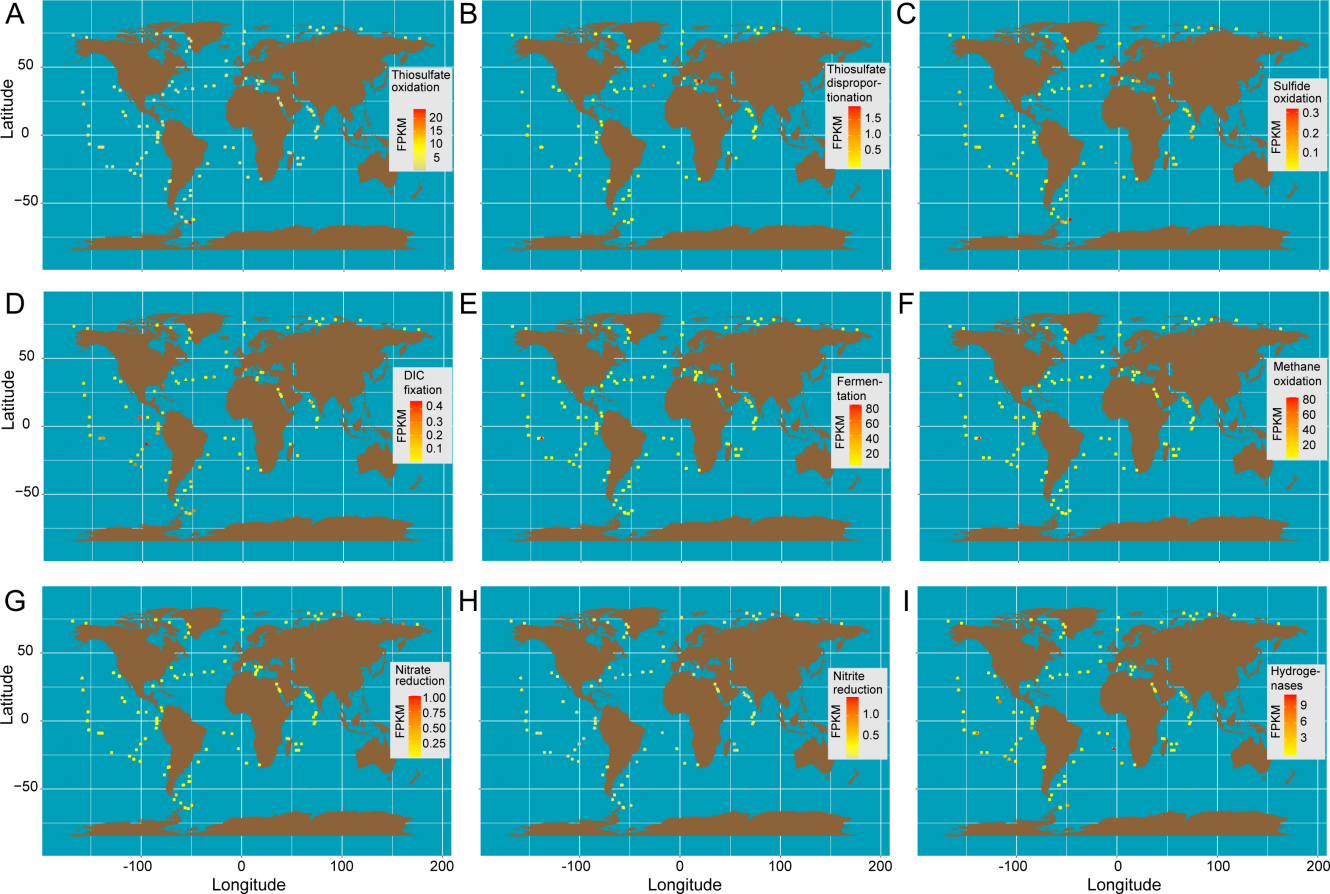
*Arcobacteraceae* transcriptional patterns in global oceans. These figures show the transcriptional abundance and distribution for select genes or metabolic pathways of *Arcobacteraceae*, including thiosulfate oxidation (**A**), thiosulfate disproportionation (**B**), sulfide oxidation (**C**), DIC fixation (**D**), fermentation (ethanol production, acetate production, and lactate production) (**E**), methane oxidation (**F**), dissimilatory nitrate reduction to nitrite (**G**), dissimilatory nitrite reduction to ammonia (**H**), and hydrogen oxidation (**I**). The transcripts from metatranscriptomic data of the Tara Oceans expeditions were calculated and normalized to fragments per kilobase of transcript sequence per million mapped reads (FPKM). The depicted shapes indicate sample sources, square for surface (5–9 m), circle for DCM, and triangle for mesopelagic; the color signifies the transcriptional abundance for each gene or metabolism.

Furthermore, the expression of genes involved in sulfur compound oxidation, DIC fixation, organic metabolism, DNRA, denitrification, and hydrogen oxidation in global oceans was confirmed, which highlights the roles of *Arcobacteraceae* in the cycling of carbon, nitrogen, and sulfur elements *in situ* (Fig. 6A through I). We found that genes in all these pathways were transcribed to varying degrees in the water columns above the mesopelagic layer (Fig. 6A through I). The active transcription of thiosulfate oxidation was found at 98% of sites, while sulfide oxidation and thiosulfate disproportionation had transcriptional activity only detected at 72% and 58% of sites, respectively (Fig. 6A through C). This indicates that thiosulfate may be the favorable inorganic energy source of *Arcobacteraceae*. Some evidence suggests that inorganic sulfur species (e.g., sulfide, thiosulfate, and sulfite) in the oxygenated water column could be produced by the degradation of dissolved organic sulfur compounds in sinking phytoplankton biomass, as well as compounds secreted by zooplankton or bacteria, such as dimethylsulfonyl propionate and taurine (33, 44–46). These sulfur compounds could be utilized by bacteria of *Arcobacteraceae* or other SOBs in the water column.

Concomitantly, DIC fixation-associated genes in *Arcobacteraceae* were transcribed to a certain extent among 80% of sites (Fig. 6D); thus, autotrophic metabolism of *Arcobacteraceae* through the oxidation of sulfur compounds was widespread in the oxygenated marine water column. In terms of organic metabolism, *Arcobacteraceae* shows transcriptional activity of fermentation (ethanol production, acetate production, and lactate production) and methane oxidation at over 98% of the sites (Fig. 6E and F). Significant difference analysis showed that the transcriptional activities of fermentation and methane oxidation are higher than those of the oxidations of sulfur compounds (ANOVA, Tukey’s test, *P* < 0.001; Table S4). These results indicate that *Arcobacteraceae* grow via a mixotrophic strategy in global marine environments and the heterotrophic metabolic activity may be slightly higher than the autotrophic metabolic activity (ANOVA, Tukey’s test, *P* < 0.001; Table S4). In contrast to that in the marine water column, the transcriptional activities of inorganic chemometabolisms (such as thiosulfate oxidation) in our DIME communities were higher than those of organic chemometabolisms, including fermentation, pyruvate oxidation, and formate oxidation (ANOVA, Tukey’s test, *P* < 0.001; Table S5). The differences in metabolic strategy (autotrophic, heterotrophic, or mixotrophic) of *Arcobacteraceae* are possibly selectively evolved mainly by the energy sources available from environmental surroundings. This is similar to nanoflagellates, in which trophic strategies are regulated by the availability of light, nutrients, and prey (9).

### Conclusion

*Arcobacteraceae* differentiated into three large clades (A, B, and C) in the phylogenomic tree, in congruence with obvious niche partitioning. Both clades A and B are limited to the terrestrial environment, while clade C is widely distributed in global oceans in addition to certain terrestrial environments, from seawater to marine sediment, and unique chemosynthetic ecosystems, such as deep-sea hydrothermal environments and whale and wood falls. The majority of *Arcobacteraceae*, regardless of phylogenetic position, possess multiple putative chemolitho-metabolic pathways in common, including those of sulfide, thiosulfate, and hydrogen oxidation for energy capture. Comparatively, clade C is more versatile in metabolism. Notably, in this clade, at least one-sixth of the members harbor the rTCA pathway for carbon fixation and are active as a mixotrophic predominant member in chemosynthetic communities in the deep sea. These results highlight the unique roles of *Arcobacteraceae* in biogeochemical cycling in marine ecosystems. Further investigations are needed to quantify their contribution *in situ* in the ocean.

## MATERIALS AND METHODS

### Incubation sample collection and description

Five incubation samples amended with wood chips, wheat bran, fish scales, fish tissue, and fish oil (DHA and EPA) were mounted on the deep-sea *in situ* microbial incubator (DIMI) in the form of a deep-sea lander and *in situ* incubated at a flat-topped seamount in the Pacific Ocean (20.4059567° N, 160.7700883° E; 1,622 m water depth) on 2 August 2017 during the 45th voyage of the Chinese Ocean. In brief, the DIMI is a self-return deep-sea microorganism *in situ* enrichment system that is deployed at the interface of deep-sea seawater and sediment, and incubations are not in contact with the sediment (47). Approximately 10 g of solid substrate or 5 mL liquid substrate was directly placed into a 50-mL tube and then mounted on the DIMI. The lander was recovered during the 50th voyage of the Chinese Ocean on 24 July 2018 (21).

### Metagenomic assembly and binning

For the metagenomic data from the five incubations and wood-fall samples (19, 48), we assembled and binned them in a previous study (30). In this study, we used the MAGs belonging to *Arcobacteraceae* for future analysis. For the 63 metagenomic data of sinking particulate organic matter collected by sediment traps at abyssal depths at ALOHA (28), we reassembled and binned them to obtain high-quality *Arcobacteraceae* MAGs. Raw metagenomic reads were subjected to quality control processing by using fastp v0.19.3 with the parameter -c (49). The 63 metagenomic data were clustered into 10 groups based on the Bray-Curtis distance of *β*-diversity, calculated using the R “vegan” package and QIIME. Filtered reads of each group were co-assembled *de novo* by MetaSPAdes v3.13.0 with the settings “-k 21,33,55” (50). The binning process was performed by using the Metawrap pipeline v1.3.2 with three methods, metabat2, maxbin2, and concoct (51). The Bin_refinement module in the Metawrap pipeline was then implemented with the parameters -c 50 and -x 10 (51). Ten binning results were combined and dereplicated using dRep v2.3.2 with the parameters -comp 50 -con 10 -sa 0.95 -g --run_tax (52). In the end, a total of 249 MAGs were obtained, of which 4 MAGs belonged to *Arcobacteraceae*. The completeness and contamination of each MAG were estimated by CheckM v1.0.12 (53). The coverage of each MAG in each sample was calculated by using Salmon software in the Metawrap pipeline with the quant_bins module (51). Taxonomic assessment of each MAG was performed using GTDBTk v2.2.3 with the database release 207 (54).

### Functional annotations

Protein-coding genes of each MAG were predicted using Prodigal v2.6.3 (55). Protein sequences were functionally annotated against databases, including KEGG (56) and eggNOG (57). The online software KAAS v2.1 (56) (https://www.genome.jp/kegg/kaas/) was used for homology searches against the KEGG database with the GHOSTZ program. EggNOG-mapper v1.0.3 software was used for annotation with the Diamond BLASTP (v0.8.36.98) method (57). When a protein sequence was annotated to the same peptidase family with the both databases, its annotation result was accepted and then used for subsequent analyses or we annotated it using NCBI’s nr database (https://blast.ncbi.nlm.nih.gov/Blast.cgi) and UniProt database (https://www.uniprot.org/) to further determine its metabolic function. Metabolic pathway analyses were determined by using the METABOLIC v2.2.3 (58). METABOLIC relies on matches to the HMM databases (KEGG KOfam, Pfam, TIGRfam, and custom HMMs) using hmmsearch implemented within HMMER to infer the presence of specific metabolic pathways in microbial genomes (58). Individual KEGG annotations are inferred in the context of KEGG modules for a better interpretation of metabolic pathways.

### Phylogenetic analysis

The phylogenomic tree for *Arcobacteraceae* was constructed based on a number of publicly available genomes (including cultured and uncultured bacteria) from NCBI, as well as MAGs from deep-sea organic enrichments, four from sediment traps, and five from wood falls, by using the UBCG method v3.0 (40). A total of 430 *Arcobacteraceae* genomes with completeness > 70% and contamination < 10% were obtained for future analysis. The completeness and contamination of each genome were estimated by CheckM v1.0.12 (53).

For the phylogenetic tree of *mmoB*, *aclY*, *SoxB*, *SoxC*, *sqr*, and *phsA* from *Arcobacteraceae* genomes in this study, along with reference proteins retrieved from the NCBI or UniProt database (https://www.uniprot.org/) were individually aligned using MUSCLE (version 3.7) (59) and then were trimmed to remove columns composed of ≥95% gaps and the taxa with <50% of the expected alignment columns using TrimAL (60). The maximum-likelihood trees were constructed using RAxML (version 8.1.24) (61), with the parameters set as “-p 12345 -m PROTGAMMALGX -x 12345 -# 100”. Then, we used the Tree Visualization By One Table (tvBOT) platform to visualize, modify, and annotate the phylogenetic trees (62).

### Metatranscriptomic mapping

The raw metatranscriptomic reads from the five DIME samples and 63 SPOM samples collected by sediment traps at abyssal depths at ALOHA (28) were subjected to quality control processing by using fastp v0.19.3 with the parameter -c (49). rRNA reads were removed by using RiboDetector v0.2.7 (63). The resulting clean reads without rRNA were mapped to MAGs using Bowtie2 with the parameters “--local -D 20 -R 3 -N 1 -L 20 -i S,1,0.50” for SPOM samples and with default parameters for five DIME samples, respectively (64). Afterward, the counting of fragments (paired-end reads) assigned to each gene was carried out using the FeatureCounts program with the parameters “-p -F GTF -g ID -t CDS -s 0 -M --fraction” (65). The transcripts were calculated and normalized to FPKM to represent the gene expression levels.

For TARA Ocean metatranscriptomic data, we used the same procedure to map reads to de-redundant genomes of clade C. One hundred forty-nine genomes of clade C were dereplicated using dRep v2.3.2 with the parameters -comp 50 -con 10 -sa 0.95 -g --run_tax (52) and produced 82 de-redundant genomes. We calculated the abundance of each genome using the standardized RPKG method mentioned in this article (8) to compare with the ubiquitous mixotrophic bacterial group UBA868 in the marine environment. The expressions of genes related to sulfur compound oxidation (*Sox*, *phsA*, and *sqr*), DIC fixation (*aclY*), organic metabolism (fermentation and methane oxidation), nitrate reduction (*napAB*), nitrite reduction to ammonia (*nirBD*), and hydrogen oxidation (hydrogenase) were calculated and normalized to FPKM.

### Global distribution pattern

To reveal the global ocean distribution pattern of *Arcobacteraceae* clade C, we mapped the transcriptional abundance of 82 de-redundant genomes on 187 of TARA Ocean stations using ggplot2 on R language v4.0. The specific script is shown in the supplemental material. Reads mapped to each genome in each sample are shown in Table S3. The transcriptional abundance (FPKM) of key metabolic pathways is shown on a world map in the same way.

### Statistical analysis

To compare the differences on the transcriptional abundance (FPKM) between autotrophic metabolism-related genes and heterotrophic metabolism-related genes in the TARA Ocean samples or in our deep-sea *in situ* organic matter enrichments and the differences on the transcriptional abundance of *Arcobacteraceae* in different water layers in the ocean, we used the ANOVA in R language v 4.0, and when the results were significant (*P* < 0.05), the post hoc Tukey HSD test (multiple comparison) was applied. The specific script is shown in the supplemental material.

To demonstrate the differences in *Arcobacteraceae* transcriptional abundance between different water layers, we plotted a box diagram using ggplot2 in R language v 4.0, and the significant differences were obtained from the post hoc Tukey HSD test. The specific script is shown in the supplemental material.

## Data Availability

The raw data including metagenomic sequences, metatranscriptomic sequences, and derived MAGs in all our DIME communities have been deposited in NODE (https://www.biosino.org/node/) with the accession numbers OEX011321, OEX011323, and OEZ007098, respectively. The 249 MAGs obtained here from the 63 metagenomic data of sinking particulate organic matter collected by sediment traps at abyssal depths at ALOHA have been deposited in NODE with the accession number OEZ014275. Five MAGs obtained here from wood falls have been deposited in NODE with the accession number OEZ014276.

## SUPPLEMENTAL MATERIAL

Supplemental material msystems.00513-24-s0001.pdf Scripts and Fig. S1-S7.

Supplemental tables msystems.00513-24-s0002.xlsx Tables S1-S5.

ASM does not own the copyrights to Supplemental Material that may be linked to, or accessed through, an article. The authors have granted ASM a non-exclusive, world-wide license to publish the Supplemental Material files. Please contact the corresponding author directly for reuse.
