# Supplementary material for: Arcobacteraceae are ubiquitous mixotrophic bacteria playing important roles in carbon, nitrogen, and sulfur cycling in global oceans
Source: mSystems. 2024 Jun 21;9(7):e00513-24. doi: 10.1128/msystems.00513-24 (PMC11265409; doi:10.1128/msystems.00513-24)
Supplement: Supplemental material — Scripts and Fig. S1-S7. [file msystems.00513-24-s0001.pdf]

Supplementary Information for

Arcobacteraceae are ubiquitous mixotrophic bacteria playing important roles in carbon, nitrogen, and sulfur cycling in global oceans

Jianyang Li[1], Shizheng Xiang[1], Yufei Li[1], Ruolin Cheng[1], Qiliang Lai[1], Liping Wang[1], Guizhen Li[1], Chunming Dong[1], Zongze Shao[1, 2] *

[1] Key Laboratory of Marine Genetic Resources of Fujian Province, Third Institute of Oceanography, Ministry of Natural Resources of PR China, Xiamen 361005, PR China;

[2] Southern Marine Science and Engineering Guangdong Laboratory (Zhuhai), Zhuhai 519000, PR China.

Running head: *Arcobacteraceae*, mixotrophic bacteria in global oceans

Keywords: *Arcobacteraceae*, mixotrophy, carbon fixation, sulfur oxidation methane oxidation, ,denitrification

*Corresponding author:

Zongze Shao. Tel: +86-592-2195321. E-mail: shaozz@163.com

Script for world map in Figures 5 and 6:

```
library(ggplot2)
library(ggmap)
library(sp)
library(maptools)
library(maps)
mp<-NULL
mapworld<-borders("world",colour = "gray90",fill="white")
mp<-ggplot()+mapworld+ylim(-90,90)
mydata<-read.table("zhuobiao.csv",header=TRUE,sep=",") # zhuobiao.csv is my data.
mp_SRF<-
mp+geom_point(aes(x=mydata$Longitude,y=mydata$Latitude,color=mydata$Mappe d_Reads.1))+
scale_color_gradient(low = "yellow",high = "red")
mp_SRF
```

Script for significant difference analysis:

```
library(multcomp)
mydata<-read.table("multcomp.csv",header=TRUE,sep=",")
Aov_comp=aov(mydata$abundance~mydata$layer)
summary(Aov_comp)
TukeyHSD(Aov_comp)
```

Script for Box plot in Figure 5D:

```
library(ggplot2)
mydata=read.table("Box_plot.csv",header=TRUE,sep=",")
Box_plot=ggplot(data=mydata,aes(x=Layer,y=Mapped_Reads,fill=Layer))+stat_boxp lot(geom = "errorbar", width=
0.1,size=0.7)+geom_boxplot(width = 0.6, outlier.shape =NA)+geom_jitter(position = position_jitter(0.2),
shape = 21, size =1)+scale_fill_manual(values = c("#00AFBB","#E7B800","
red"))+theme_bw(base_size = 15)+theme(legend.position ="none")+ylim(0,5)
Box_plot
```

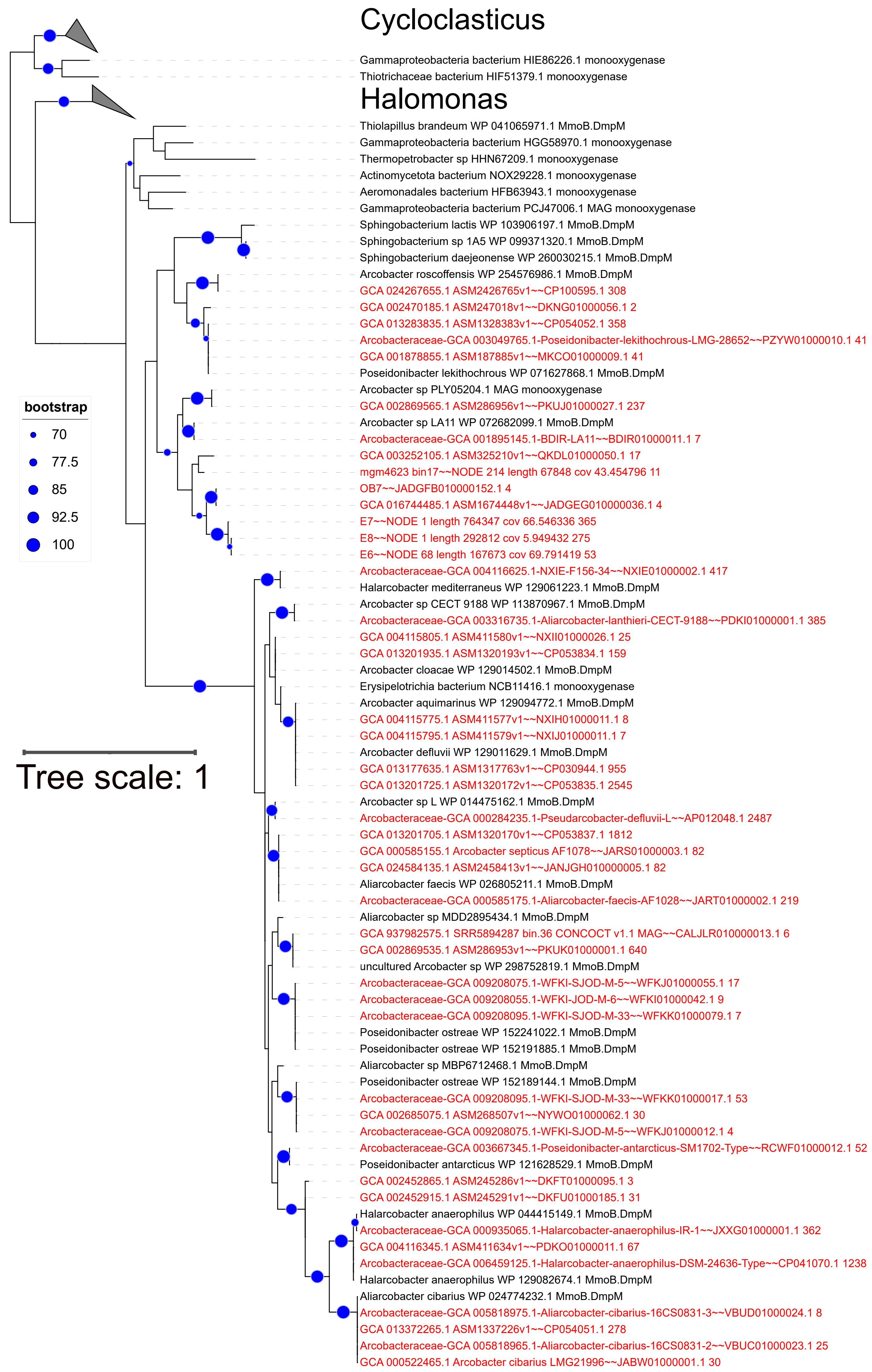

Fig. S1 Phylogenetic reconstruction of mmoB peptides from Arcobacteraceae and representatives from other groups. 13 of 143 in A group, 1 of 138 in B group, and 38 of 149 in C group were predicted to harbor mmoB, respectively, which is putatively involved in methane oxidation. Peptide sequences were aligned with MUSCLE and poorly aligned positions in the alignment were removed with TrimAL. The tree was built with RaxMLHPC-PTHREADS-SSE3 with the parameters set as “-p 12345 -m PROTGAMMALGX-x 12345 -# 100”. Genes derived in this study are shown in red, and others were retrieved from the UniProt database (https://www.uniprot.org/).

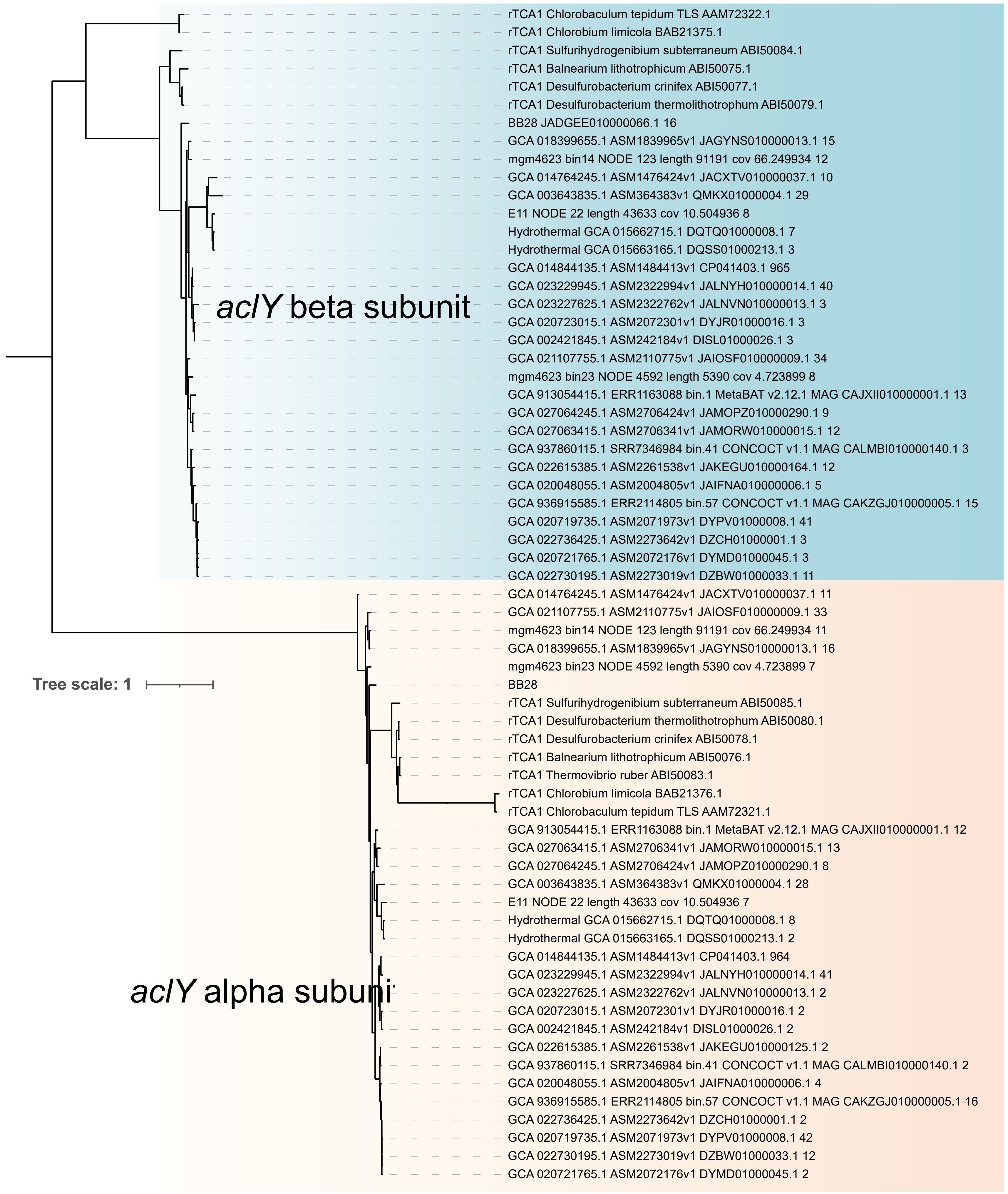

Fig. S2 Phylogenetic reconstruction of aclY alpha- and beta- subunit peptides from Arcobacteraceae and representatives from other groups. Out of 149 group C genomes 26 were predicted to harbor aclY , which is putatively involved in the reductive tricarboxylic acid (rTCA) cycle for DIC fixation. Peptide sequences were aligned with MUSCLE and poorly aligned positions in the alignment were removed with TrimAL.The tree was built with RaxMLHPC-PTHREADS-SSE3 with the parameters set as “-p 12345 -m PROTGAMMALGX -x 12345 -# 100”.

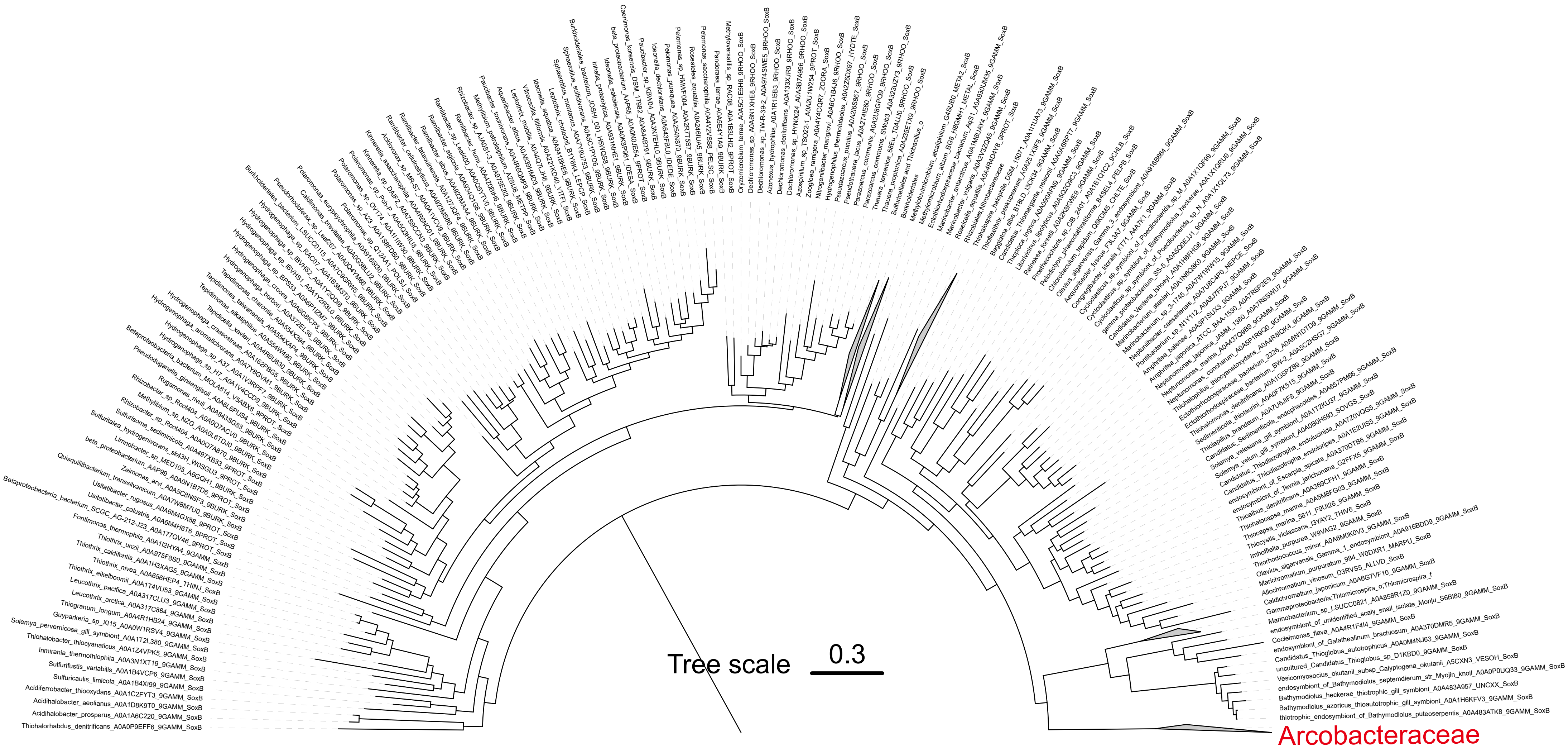

Fig. S3 Phylogenetic reconstruction of SoxB peptides from Arcobacteraceae and representatives from other groups. 142 of 143 in A group, 9 of 138 in B group, and 114 of 149 in C group were predicted to harbor SoxB, respectively, which is putatively involved in thiosulfate oxidation. Peptide sequences were aligned with MUSCLE and poorly aligned positions in the alignment were removed with TrimAL.The tree was built with RaxMLHPC-PTHREADS-SSE3 with the parameters set as “-p 12345 -m PROTGAMMALGX -x 12345 -# 100”.

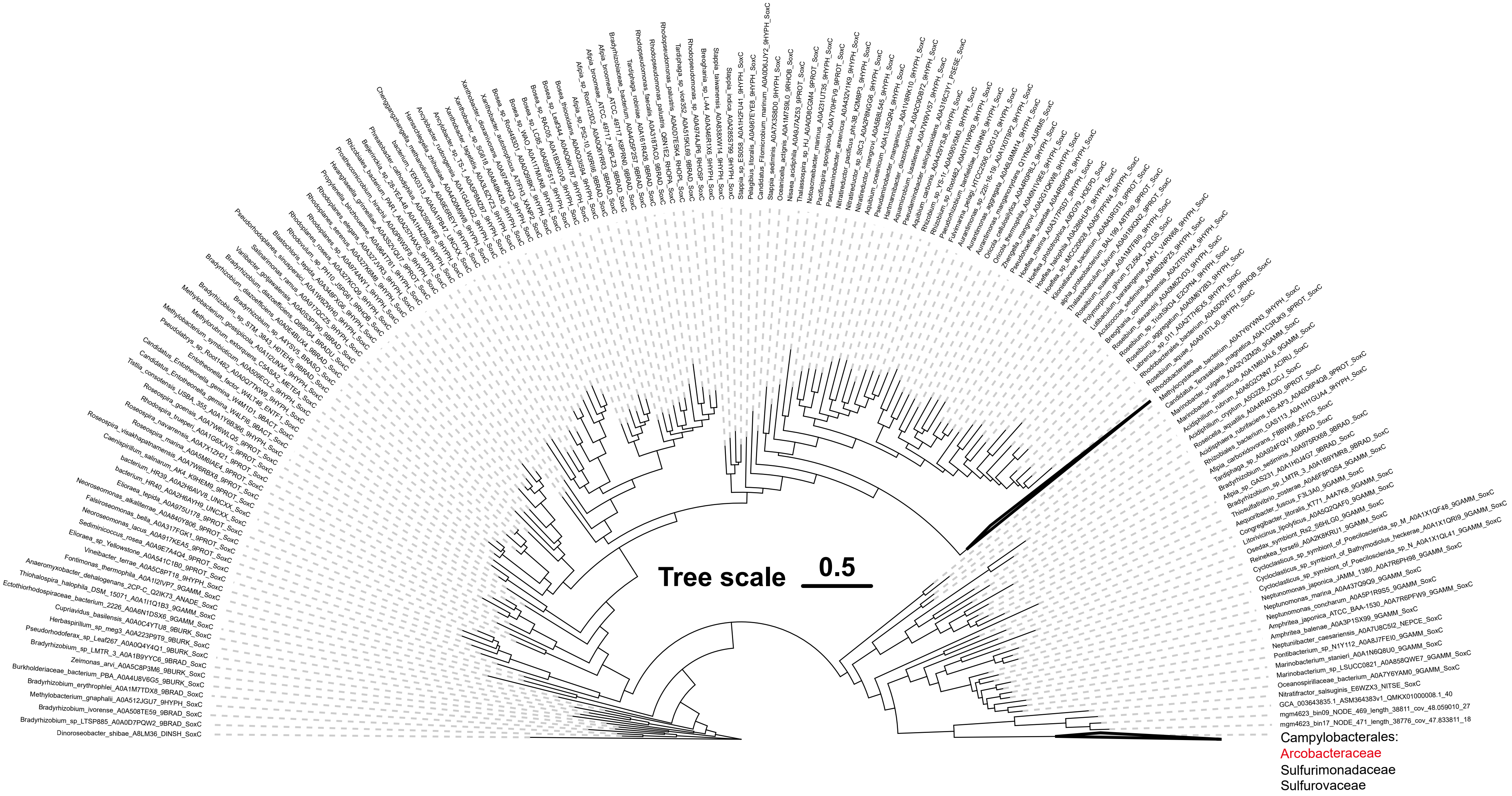

Fig. S4 Phylogenetic reconstruction of SoxC peptides from Arcobacteraceae and representatives from other groups. 142 of 143 in A group, 9 of 138 in B group, and 114 of 149 in C group were predicted to harbor SoxC, respectively, which is putatively involved in thiosulfate oxidation. Peptide sequences were aligned with MUSCLE and poorly aligned positions in the alignment were removed with TrimAL. The tree was built with RaxMLHPC-PTHREADS-SSE3 with the parameters set as “-p 12345 -m PROTGAMMALGX -x 12345 -# 100”.

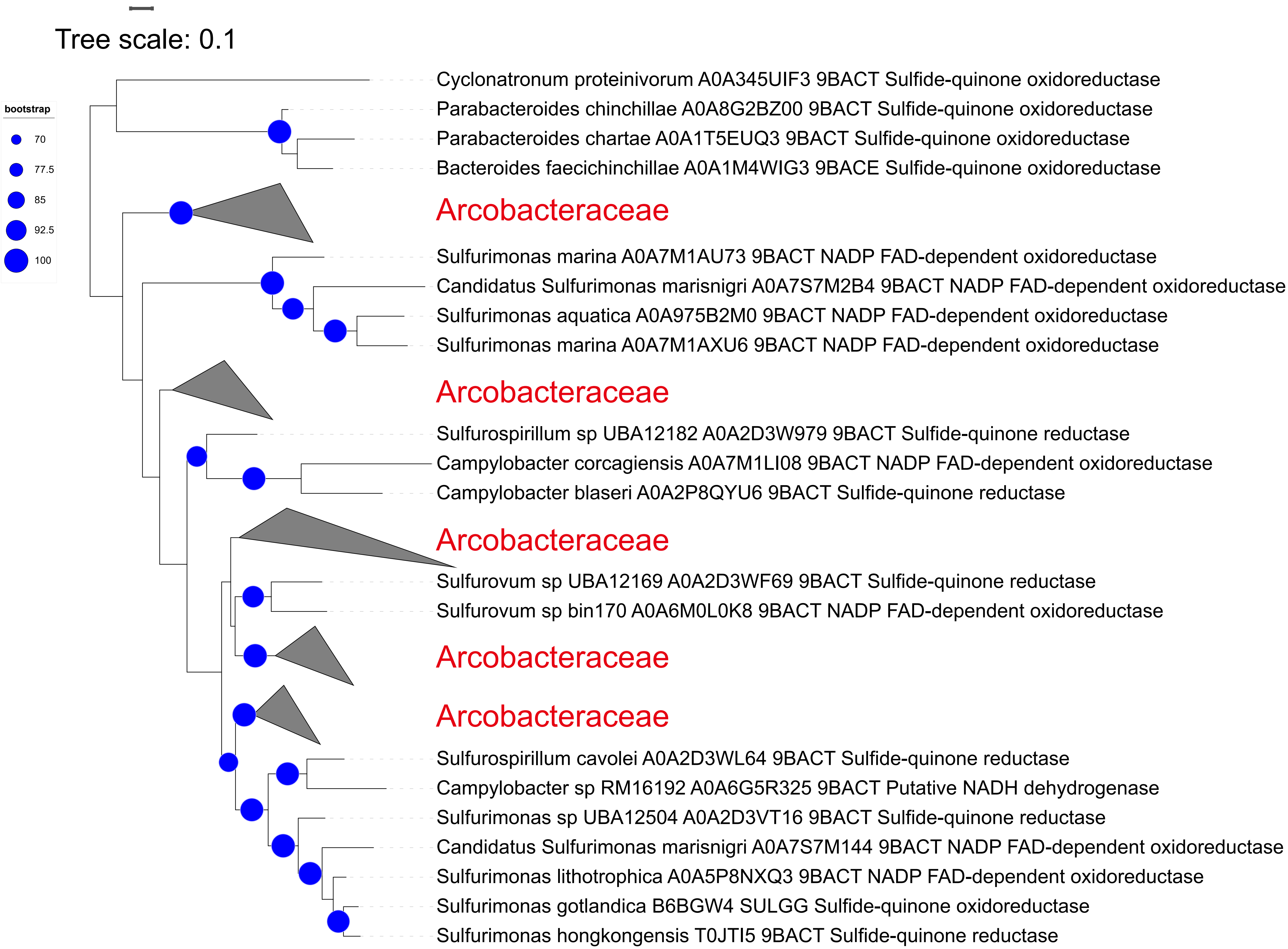

Fig. S5 Phylogenetic reconstruction of sqr peptides from Arcobacteraceae and representatives from other groups. 142 of 143 in A group, 133 of 138 in B group, and 121 of 149 in C group were predicted to harbor sqr, respectively, which is putatively involved in sulfide oxidation. Peptide sequences were aligned with MUSCLE and poorly aligned positions in the alignment were removed with TrimAL. The tree was built with RaxMLHPC-PTHREADS-SSE3 with the parameters set as “-p 12345 -m PROTGAMMALGX -x 12345 -# 100”. Genes derived in this study are shown in red, and others were retrieved from the UniProt database (https://www.uniprot.org/).

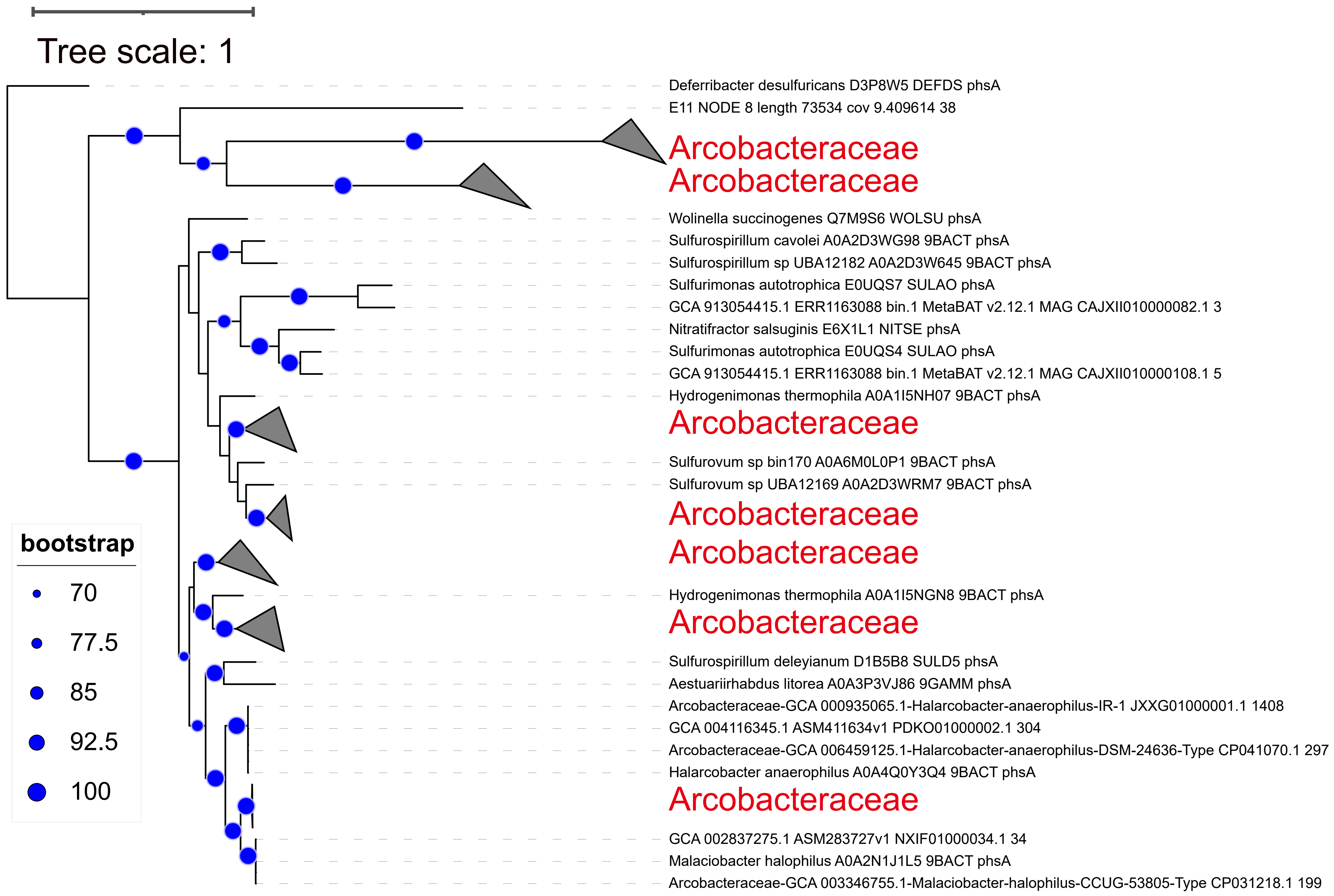

Fig. S6 Phylogenetic reconstruction of phsA peptides from Arcobacteraceae and representatives from other groups. Only 31 of 149 in C group were predicted to harbor phsA, which is putatively involved in thiosulfate disproportionation. Peptide sequences were aligned with MUSCLE and poorly aligned positions in the alignment were removed with TrimAL. The tree was built with RaxMLHPC-PTHREADS -SSE3 with the parameters set as "-p 12345 -m PROTGAMMALGX -x 12345 -# 100". Genes derived in this study are shown in red, and others were retrieved from the UniProt database (https://www.uniprot.org/).

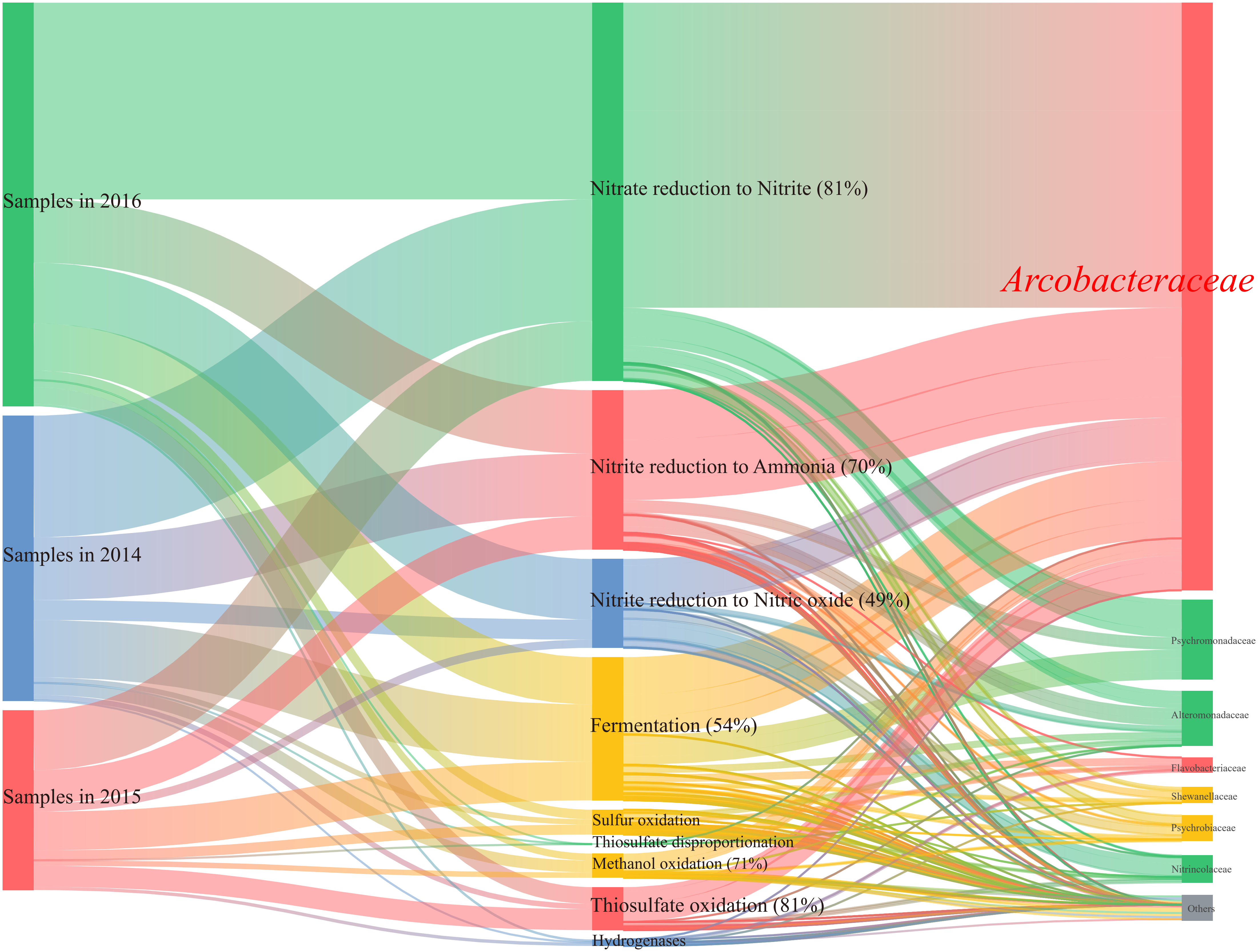

Fig. S7 Transcriptional profiles of preponderant Arcobacteraceae in the sinking particulate organic matter from the ALOHA abyssal depths. The percentage in brackets refers to the proportion of Arcobacteraceae in the corresponding metabolic transcript.
